# Differential MicroRNA Expression in the Anterior Lens Capsule of Patients with Glucocorticoid-Induced Cataracts: A Preliminary Study

**DOI:** 10.3390/jcm14196909

**Published:** 2025-09-29

**Authors:** Yeji Yeon, Soo Rack Ryu, Min-Ji Cha, Won June Lee, Han Woong Lim, Ji Hong Kim, Yu Jeong Kim

**Affiliations:** 1 Department of Ophthalmology, Hanyang University College of Medicine, Seoul 04763, Republic of Korea; neumond@hanyang.ac.kr (Y.Y.); minjicha619@gmail.com (M.-J.C.); wonjunelee@hanyang.ac.kr (W.J.L.); hwlim@hanyang.ac.kr (H.W.L.); 2 Hanyang Vision Research Center, Hanyang University, Seoul 04763, Republic of Korea; 3 Biostatistical Consulting and Research Laboratory, Medical Research Collaborating Center, Hanyang University, Seoul 04763, Republic of Korea; rsa4648@hanyang.ac.kr; 4 Department of Ophthalmology, Hanyang University Seoul Hospital, Seoul 04763, Republic of Korea; 5 Hanyang Institute of Bioscience and Biotechnology, Hanyang University, Seoul 04763, Republic of Korea

**Keywords:** microRNA, glucocorticoid-induced cataract, posterior subcapsular cataract, oxidative stress, lens epithelial cells

## Abstract

**Background/Objectives**: To investigate microRNA (miRNA) expression profiles in the anterior lens capsules of patients with glucocorticoid-induced cataracts (GIC) and to identify miRNAs potentially associated with glucocorticoid (GC) exposure and posterior subcapsular cataract (PSC) formation. **Methods**: A total of 33 participants were divided into four groups based on their GC usage history and cataract type: GIC (*n* = 10), age-related PSC (*n* = 6), GC-treated age-related cataract (ARC) (*n* = 7), and normal control (*n* = 10). Anterior lens capsule samples were obtained during cataract surgery and total RNA was extracted for quantitative real-time polymerase chain reaction (qRT-PCR). The expression levels of 12 selected miRNAs were quantified using a customized miScript miRNA PCR array. **Results**: Among the twelve miRNAs analyzed, seven (let-7a-5p, let-7d-5p, miR-15a-5p, miR-16-5p, miR-23b-3p, miR-26a-5p, and miR-125a-5p) were significantly differentially expressed among the groups (*p* < 0.05). In the GIC group, let-7a-5p, miR-23b-3p, miR-26a-5p, and miR-125a-5p were significantly upregulated, whereas let-7d-5p, miR-15a-5p and miR-16-5p were significantly downregulated compared to that in the normal control group. No significant differences in miRNA expression were observed between the GIC and age-related PSC groups or between the GIC and GC-treated ARC groups. **Conclusions**: This study demonstrates distinct miRNA expression patterns in the anterior lens capsules of patients with GIC. Altered expression of specific miRNAs may be linked to the pathogenesis of GC-induced PSC formation. These findings provide a molecular basis for further investigation into the regulatory roles of miRNAs in GC-associated cataracts.

## 1. Introduction

Cataracts, characterized by lens opacity leading to impaired vision, are a major global cause of blindness [1]. Depending on the etiology, cataracts are classified into age-related, pediatric, and secondary forms [2]. Age-related cataracts (ARCs) are further subdivided into nuclear, cortical, and posterior subcapsular cataracts (PSC), based on the location of opacity [3]. PSCs are particularly associated with migration and abnormal proliferation of lens epithelial cells (LECs) beneath the posterior capsule and account for a significant proportion of visually disabling cataracts in both age-related and secondary forms.

Secondary cataracts can arise from various factors, including trauma, radiation, metabolic disorders, and medication use—particularly corticosteroids [2,4]. Glucocorticoids (GCs), cholesterol-derived compounds, are potent anti-inflammatory and immunosuppressive agents widely used in the treatment of inflammatory, allergic, autoimmune, and immunologic conditions [5,6,7]. However, prolonged systemic GC use can result in adverse effects, including ocular complications such as cataracts, particularly PSC or glucocorticoid-induced cataracts (GIC) [7,8,9,10]. Despite evidence linking GCs to GIC pathogenesis, the underlying molecular mechanisms remain incompletely understood.

MicroRNAs (miRNAs) are small endogenous non-coding RNAs that regulate gene expression post-transcriptionally and are critical in cellular processes such as differentiation, proliferation, and apoptosis [11,12,13,14]. While extensive research has investigated the role of miRNAs in various eye diseases [15,16,17], our previous study focused on miRNA expression in the anterior lens capsules of patients with senile cataracts categorized by cataract type [18]. However, reports on miRNA expression in the anterior lens capsules of patients with GIC remain scarce.

To explore this, we focused on anterior lens capsule tissue, which is ethically and surgically accessible during routine phacoemulsification via continuous curvilinear capsulorhexis. This tissue contains LECs, which are central to the pathogenesis of PSC and respond to glucocorticoid signaling and oxidative stress. Compared to aqueous humor or systemic samples, the anterior capsule provides a more direct lens-specific biological context for evaluating cataract-related molecular alterations.

We hypothesized that patients with GIC exhibit distinct miRNA expression profiles in the anterior lens capsule compared to other types of cataract or healthy controls, reflecting steroid-induced molecular dysregulation. To test this hypothesis, we analyzed the differences in miRNA expression between patients with GIC and comparison groups based on lens opacity location and history of GC use. Additionally, among GC users, we examined the correlations between GC use duration, daily dose, cumulative dose, and cataract formation.

## 2. Materials and Methods

### 2.1. Study Participants

This study was approved by the Hanyang University Institutional Human Experimentation Committee (IRB number: HYUH 2018-10-013-003) and was conducted in strict compliance with the principles outlined in the Declaration of Helsinki. Written informed consent for the use of human tissue samples was obtained from all participants. All participants underwent a comprehensive preoperative ophthalmologic examination, including best-corrected visual acuity (BCVA) assessment, intraocular pressure measurement, automated refraction, fundus examination, and slit-lamp examination. Lens opacities were classified as cortical, nuclear, or PSCs using the Lens Opacities Classification System (LOCS) III during slit-lamp examination. Patients with a history of ocular surgery, trauma, intraocular inflammation, diabetes, or radiation exposure were excluded.

The participants were divided into four groups based on age, history of GC use, and presence of PSC.

Group 1 (GIC group): Participants with past or current GC use who presented with PSC-type cataracts under the age of 60 years.

Group 2 (Age-related PSC group): Participants with PSC cataracts aged ≥ 60 years but with no history of GC use.

Group 3 (GC-treated ARC group): Participants with non-PSC-type cataracts aged ≥ 60 years and with past or current GC use.

Group 4 (Normal control group): Participants under age 60 years but with no history of GC use and minimal cataracts (≤LOCS grade 1), who underwent clear lens extraction for the insertion of a multifocal intraocular lens for presbyopia correction.

The duration of GC use and cumulative GC dose were investigated for patients in Groups 1 and 3, and the daily GC intake was calculated by dividing the cumulative GC dose by the duration of GC use.

### 2.2. Sample Collection

The method for acquiring anterior lens capsules has been previously described [18]. All participants underwent phacoemulsification, followed by insertion of a posterior chamber intraocular lens. During the continuous curvilinear capsulorhexis procedure, anterior lens capsules were obtained with diameters of approximately 5–6 mm and centered on the lens. The collected samples were placed in microtubes and immediately frozen at −80 °C for subsequent analysis.

### 2.3. Total RNA Extraction, Complementary DNA (cDNA) Synthesis, and Real-Time Polymerase Chain Reaction

The methods for total RNA extraction, complementary DNA (cDNA) synthesis, and quantitative real-time polymerase chain reaction (qRT-PCR) have been previously described. Lens capsule samples were homogenized in QIAzol reagent (Qiagen, Valencia, CA, USA) to extract total RNA, including small RNAs and miRNAs. RNA was isolated using a miScript Micro Kit (Qiagen) according to the manufacturer’s instructions. The quality of the extracted RNA was evaluated by measuring the OD260/280 ratio using a spectrophotometer (NanoDrop ND-2000; Thermo Fisher Scientific, Waltham, MA, USA). All RNA samples were stored at −80 °C until use in cDNA synthesis. For reverse transcription into cDNA, the miScript II RT Kit (Qiagen) was used, following the manufacturer’s protocol. A customized miScript miRNA-PCR array was used to profile the expression of 12 miRNAs of interest: let-7a-5p, let-7d-5p, let-7g-5p, miR-15a-5p, miR-16-5p, miR-22-3p, miR-23a-3p, miR-23b-3p, miR-26a-5p, miR-34a-5p, miR-125a-5p, and miR-125b-5p. These 12 miRNAs were selected based on our previous study investigating miRNA profiles in senile cataracts with different morphological subtypes [18]. qRT-PCR was performed using SYBR Green-based RT-PCR on a Roche LightCycler 480 system (Roche, Basel, Switzerland), in accordance with the manufacturer’s guidelines.

### 2.4. Normalization and Relative Quantification of Anterior Lens Capsule miRNA Ex-Pression

To address the normalization challenges associated with miRNA expression in the anterior lens capsules, particularly due to the absence of stable RNA, the global mean normalization method was used, as described in previous studies [19]. Tissue miRNA expression data were normalized by calculating the mean Ct value using SNORD96A as the reference gene, using the Gene Global Data Analysis Center (Qiagen).

The relative expression of miRNAs was determined using the comparative 2^−ΔΔCt^ method. Fold change was calculated using the equation 2^−ΔΔCt^, where ΔCt = Ct (target gene)—Ct (reference gene) [20,21]. To evaluate differences in miRNA expression among the groups, the fold regulation value was employed, calculated as the reciprocal of the negative fold change value.

### 2.5. Statistical Analysis

The data were expressed as means ± standard deviation (SD). To address significant age differences between groups, the inverse probability of treatment weighting (IPTW) method was applied to mitigate age-related effects. Differences between the two groups were assessed using the chi-square test for categorical variables and Student’s *t*-test for continuous variables. One-way analysis of variance (ANOVA) was used for comparisons among the three groups. Correlations between miRNA expression and GC intake parameters, including duration of GC use, cumulative GC dose, and daily GC intake, were evaluated using Pearson’s correlation coefficients for the GIC and GC-treated ARC groups. Statistical analyses were performed using SAS version 9.4 software (SAS Institute Inc., Cary, NC, USA), with statistical significance set at *p* < 0.05.

## 3. Results

### 3.1. Demographic and Clinical Characteristics of the Study Population

Table 1 summarizes the demographic and clinical characteristics of the four groups: the GIC group (*n* = 10), the age-related PSC group (*n* = 6), the GC-treated ARC group (*n* = 7), and the normal control group (*n* = 10). Prior to IPTW, the mean age of the GIC group was 48.80 ± 13.90 years, which was significantly lower than that of the other groups: 64.67 ± 6.19 years in the age-related PSC group, 62.84 ± 7.54 years in the GC-treated ARC group, and 61.00 ± 4.83 years in the normal control group (*p* = 0.005). After IPTW adjustment, the age differences were no longer statistically significant (*p* = 0.353). Sex distribution also showed significant differences across the groups, with all participants in the GIC and normal control groups being female, whereas the GC-treated ARC and age-related PSC groups included both sexes (*p* = 0.002 before IPTW). The BCVA (logMAR) differed significantly among the groups, with the GIC group showing intermediate vision (0.56 ± 0.51), compared to 0.26 ± 0.14 in the GC-treated ARC group, 0.82 ± 0.59 in the age-related PSC group, and 0.00 ± 0.01 in the normal control group (*p* = 0.001 before IPTW). Refractive error, expressed as spherical equivalent (SE), showed no significant difference before IPTW (*p* = 0.617); however, it reached statistical significance after adjustment (*p* = 0.040).

### 3.2. Expression of MicroRNAs in Patients with GIC, Age-Related PSC, GC-Treated ARC, and Normal Controls

Table 2 shows the expression profiles of the 12 selected miRNAs across the four groups. Seven miRNAs showed statistically significant differences in expression between the groups: let-7a-5p, let-7d-5p, miR-15a-5p, miR-16-5p, miR-23b-3p, miR-26a-5p, and miR-125a-5p (all *p* < 0.05). Compared with that in the control group, the GIC group exhibited significant upregulation of let-7a-5p (*p* < 0.001), miR-23b-3p (*p* = 0.017), miR-26a-5p (*p* < 0.001), and miR-125a-5p (*p* = 0.010), whereas let-7d-5p (*p* = 0.015), miR-15a-5p (*p* = 0.005), and miR-16-5p (*p* = 0.001) were significantly downregulated. Notably, let-7a-5p showed the greatest increase and miR-15a-5p showed the greatest decrease in expression in the GIC group compared to that in the normal controls. No significant differences in miRNA expression were observed between GIC and age-related PSC groups. Similarly, comparison between the GIC- and GC-treated ARC groups did not reveal statistically significant differences in the expression of any of the miRNAs.

**Figure 1:**
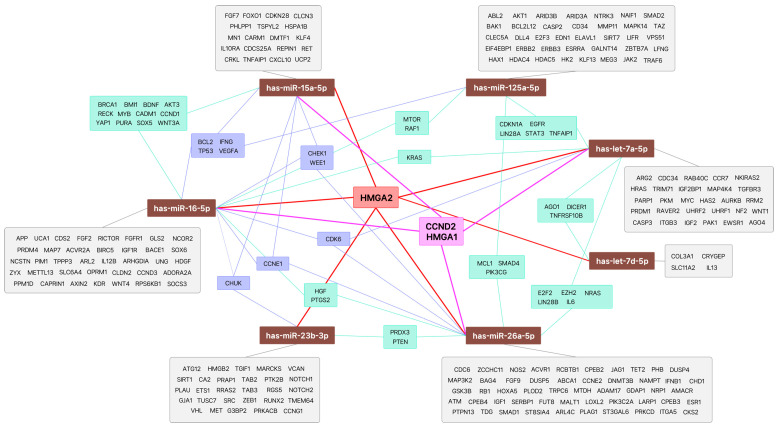
Predicted target genes of differentially expressed miRNAs in glucocorticoid-induced cataract using bioinformatics analysis.

Bioinformatics analysis of genes associated with the seven miRNAs was performed using miRTargetLink 2.0 (https://ccb-compute.cs.uni-saarland.de/mirtargetlink2/ accessed on 8 August 2024), which identified several potential target genes, including HMGA2, HMGA1, and CCND2 (Figure 1).

### 3.3. Correlation Between Clinical Parameters and miRNA Expression in GIC

Table 3 shows the results of correlation analyses between clinical variables and miRNA expression in the GIC group. Among the 12 miRNAs analyzed, miR-23a-3p showed a statistically significant positive correlation with daily GC intake (r = 0.639, *p* = 0.047). Additionally, miR-125a-5p expression was significantly correlated with daily GC intake (r = 0.688, *p* = 0.028). No significant correlations were found between miRNA expression and patient age or cumulative GC dose for any miRNA.

## 4. Discussion

PSCs are characterized by the migration of equatorial LECs toward the posterior pole of the lens, where they accumulate and form opacities under the posterior capsule. PSCs account for approximately 10% of all senile cataracts and are associated with various risk factors, including atopy, diabetes mellitus, hypoparathyroidism, ocular inflammation, trauma, ionizing radiation, myopia, ultraviolet radiation, steroids, and prior vitrectomy procedures [22,23,24]. Among these, GC use is a well-established contributor to PSC formation, although the precise molecular mechanisms of steroid-induced cataractogenesis remain incompletely understood. The proposed mechanisms include increased intraocular glucose levels, oxidative stress, and reduced lenticular glutathione levels [25]. Additionally, GCs are known to interfere with the proliferation and differentiation of normal LECs via GC receptors, potentially reducing cell adhesion molecules, such as E-cadherin, and activating signaling pathways involving fibroblast growth factors [9].

In this study, we investigated the miRNA expression profiles in the anterior lens capsules of patients with GIC and compared them with those of patients with age-related PSCs, patients with GC-treated ARC, and normal controls. The selected 12 miRNAs were chosen based on their established or hypothesized roles in key biological processes involved in cataractogenesis, such as oxidative stress, apoptosis, and cell cycle regulation. Prior literature has implicated miR-15a, miR-16, and miR-125a in apoptotic signaling; miR-23b and miR-26a in oxidative stress responses; and members of the let-7 family in epithelial cell differentiation and senescence, as discussed in detail in the subsequent sections. Importantly, the same panel was previously utilized in our earlier study examining miRNA profiles across morphological subtypes of senile cataracts, enabling meaningful comparisons and methodological consistency across cataract types [18].

**Figure 2:**
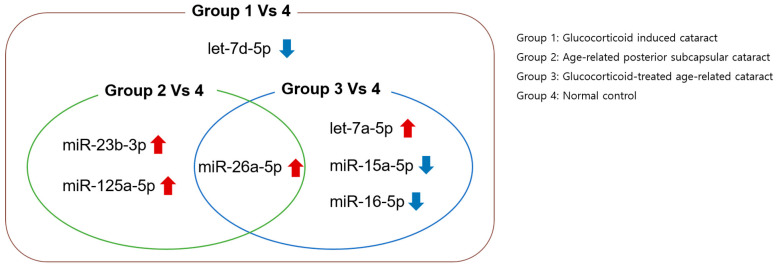
Venn diagram illustrating differentially expressed miRNAs in glucocorticoid-induced cataracts compared with normal controls and overlap with other cataract groups. Red upward arrows indicate significantly upregulated miRNAs compared to the control group, whereas blue downward arrows indicate significantly downregulated miRNAs.

We identified seven miRNAs—let-7a-5p, let-7d-5p, miR-15a-5p, miR-16-5p, miR-23b-3p, miR-26a-5p, and miR-125a-5p—that were differentially expressed in the GIC group compared to those in normal controls. Interestingly, all these miRNAs, except let-7d-5p, were also differentially expressed in GC-treated ARC or age-related PSCs, suggesting that GIC represents a convergence of miRNA dysregulation related to both GC exposure and PSC-type cataractogenesis (Figure 2).

Among the steroid-related miRNAs, miR-26a-5p was notably upregulated not only in the GIC group but also in other GC-related cataract groups, suggesting a consistent association with GC exposure. Previous studies have demonstrated increased miR-26a-5p expression in models of steroid-induced osteonecrosis of the femoral head, where long-term GC use alters bone metabolism and vascular integrity [26]. These findings imply that miR-26a-5p may be responsive to steroid-mediated metabolic disturbances, particularly those involving calcium balance, vitamin D signaling, and growth factor regulation. These pathways are relevant to lens physiology. For instance, calcium and vitamin D play key roles in maintaining lens transparency, and their dysregulation has been implicated in cataract formation [27,28]. GCs interfere with these systems, potentially contributing to biochemical stress within the lens. Furthermore, GCs modulate growth factors such as fibroblast growth factors (FGFs) and transforming growth factor-beta (TGF-β), which influence lens epithelial cell behavior, including proliferation, differentiation, and extracellular matrix turnover [8]. The upregulation of miR-26a-5p in our study may therefore reflect a lens-specific response to these systemic and local changes induced by chronic GC exposure. miR-26a-5p may act as a molecular link between altered systemic metabolism and lens epithelial dysfunction, ultimately promoting PSC development.

Conversely, miR-15a-5p and miR-16-5p were significantly downregulated in the GIC group, in contrast to that in previous reports. For instance, the upregulation of miR-15a and miR-16 has been reported in eyes treated with intravitreal triamcinolone acetonide [29], and an increase in circulating miR-15a was observed following the systemic administration of dexamethasone [30]. These discrepancies may be attributed to differences in sample type, route, and duration of GC exposure, or time points at which samples were collected post-treatment. Moreover, tissue-specific miRNA regulation is increasingly recognized as a major factor influencing miRNA dynamics, with the same miRNA exhibiting opposing trends in different organ systems or cell types. The miR-15/16 cluster is known to function as a critical regulator of apoptosis and cell cycle progression, often acting by targeting anti-apoptotic genes such as BCL2 [29]. In immune cells, GCs induce the overexpression of this cluster, thereby promoting apoptosis and facilitating the resolution of inflammation [31]. In contrast, our findings of reduced miR-15a-5p and miR-16-5p expression in the lens epithelium under chronic GC exposure may reflect a lens-specific protective adaptation to long-term steroid stress, possibly aimed at suppressing excessive cell death in the avascular lens tissue. Alternatively, this downregulation may represent a maladaptive process that contributes to abnormal LEC proliferation or impaired apoptosis, both of which have been implicated in the formation of PSCs.

With regard to oxidative stress, miR-23b-3p and miR-125a-5p were significantly upregulated in the GIC and age-related PSC groups, suggesting a shared role in oxidative stress-related cataractogenesis. Oxidative damage has long been recognized as a key contributor to cataract formation, particularly through mechanisms such as reactive oxygen species (ROS)-induced apoptosis, protein aggregation, and LEC damage. Reportedly, miR-23b-3p expression increases in LECs under hydrogen peroxide-induced oxidative stress, and its knockdown reduces apoptosis while increasing autophagy, indicating its potential role as a stress-responsive regulator in lens cells [32,33]. Similarly, miR-125a-5p responds to oxidative conditions, and its expression increases in mesenchymal stem cells exposed to ROS [34]. Notably, miR-125a-5p is known to regulate genes associated with cell cycle arrest and apoptosis, including p53, p21, and p16, which may have important implications for lens epithelial cell survival and cataract pathogenesis under oxidative stress. These findings suggest that miR-23b-3p and miR-125a-5p may serve not only as molecular indicators of redox imbalance in the lens microenvironment but also as active mediators of oxidative injury, contributing to posterior subcapsular opacity and cataract formation, particularly under conditions exacerbated by GC exposure.

Additionally, let-7a-5p expression was upregulated, whereas let-7d-5p expression was downregulated in the GIC group. These two miRNAs belong to the let-7 family, which is widely recognized for its roles in tumor suppression and, in certain contexts, tumor promotion [35,36]. In addition, let-7a-5p reportedly inhibits cell proliferation, migration, and epithelial-mesenchymal transition, whereas let-7d-5p is involved in modulating apoptosis, inflammation, and cellular stress responses. However, despite their well-documented roles in oncogenesis and cell regulation, current evidence of their association with steroid signaling or GC-induced tissue remodeling remains limited. Given the distinct expression patterns observed in our study and their known regulatory functions in other biological systems, further investigations are warranted to elucidate whether these miRNAs contribute to the pathogenesis of GC-induced cataracts by modulating oxidative stress, lens epithelial cell dynamics, or fibrotic remodeling in the lens microenvironment under chronic steroid exposure.

In our correlation analyses, we observed that miR-23a-3p and miR-125a-5p expression levels were significantly associated with daily GC intake in patients with GIC, whereas no consistent relationships were identified with cumulative dose or duration of GC use. These findings suggest that the intensity of daily glucocorticoid exposure may exert a more immediate influence on lens epithelial cell homeostasis than the total lifetime dose. Mechanistically, miR-23a-3p has been implicated in oxidative stress-induced apoptosis, while miR-125a-5p is known to regulate cell cycle and stress-response pathways, both of which are relevant to PSC development. The absence of significant correlations with cumulative dosage might be explained by interindividual variability in steroid responsiveness, differences in treatment regimens, or the relatively small sample size of our cohort. Nonetheless, the positive association with daily intake highlights the potential of these miRNAs as indicators of acute steroid-induced stress in the lens epithelium.

This study has some limitations. First, the sample size was relatively small, which may have reduced the statistical power and limited the generalizability of our findings. Second, only a predefined panel of 12 miRNAs was analyzed, potentially overlooking other relevant regulatory RNAs involved in GC-induced cataractogenesis. Third, although we identified differentially expressed miRNAs, we did not perform functional validation experiments such as gene target analysis or in vitro overexpression/inhibition assays to confirm their biological roles. Fourth, although patients were categorized based on GC use and cataract type, there was variability in the GC formulation, route, dosage, and duration of exposure, which could have influenced miRNA expression. In addition, our analyses were limited to the anterior lens capsule tissue, which may not fully reflect the molecular changes occurring in deeper lens layers. However, the anterior capsule is the portion routinely obtained during standard phacoemulsification via continuous curvilinear capsulorhexis, making it ethically and practically suitable for research use. Moreover, this layer contains LECs, which are central to the pathophysiology of posterior subcapsular cataract development, particularly in response to glucocorticoid exposure and oxidative stress. Thus, while deeper lens regions were not directly assessed, our tissue selection remains biologically and clinically relevant. Finally, the cross-sectional design of the study precludes conclusions regarding the causality between GC exposure, changes in miRNA expression, and cataract development.

Future studies should include functional validation using human LEC models treated with glucocorticoids, combined with overexpression or knockdown of candidate miRNAs, to confirm downstream target interactions. In addition, lens organoid systems or in vivo models may help clarify the mechanistic roles of these miRNAs in PSC development under steroid exposure. If validated consistently, such miRNAs could serve as early biomarkers of lens epithelial stress or dysregulation in patients receiving glucocorticoids, enabling earlier identification of individuals at risk for PSC formation. In the longer term, pharmacologic modulation of these miRNAs—via antagomirs or mimics—may offer a targeted therapeutic strategy to prevent or delay steroid-induced cataractogenesis.

## 5. Conclusions

In conclusion, our study demonstrates that GIC is associated with distinct miRNA expression profiles in the anterior lens capsule, reflecting a combination of molecular alterations related to both steroid exposure and PSC pathogenesis. Notably, miR-23b-3p, miR-26a-5p, and miR-125a-5p were upregulated in association with oxidative stress, whereas miR-15a-5p and miR-16-5p were downregulated, suggesting the disruption of apoptosis and cellular homeostasis. Concurrent upregulation of let-7a-5p and downregulation of let-7d-5p may represent GIC-specific regulatory features. These findings provide preliminary evidence for the role of miRNAs in steroid-related lens changes and offer insights into the potential molecular pathways that contribute to GIC development. Further research is needed to validate these observations and explore their therapeutic relevance in the prevention and management of GICs.

## Figures and Tables

**Table 1:** Demographics and clinical characteristics of the study participants.

	Before IPTW					After IPTW				
	Group ^†^ 1	Group 2	Group 3	Group 4	*p*	Group 1	Group 2	Group 3	Group 4	*p*
Age (years)	48.80 ± 13.90	64.67 ± 6.19	62.84 ± 7.54	61.00 ± 4.83	0.005 *	57.68 ± 10.48	62.75 ± 4.64	61.61 ± 6.69	60.68 ± 4.72	0.353
Sex (No. Female/Male)	10/0	2/4	5/2	10/0	0.002 *	10/0	1/3	5/2	9/0	0.003 *
BCVA (logMAR)	0.56 ± 0.51	0.82 ± 0.59	0.26 ± 0.14	0.00 ± 0.01	0.001 *	0.53 ± 0.37	0.81 ± 0.50	0.26 ± 0.12	0.00 ± 0.01	0.005 *
SE (diopters)	−0.39 ± 2.86	0.04 ± 2.22	0.04 ± 2.22	0.53 ± 1.23	0.617	−2.52 ± 3.12	−0.39 ± 1.77	−0.71 ± 1.54	0.55 ± 1.13	0.040 *
Duration of GC Intake (years)	8.60 ± 5.91		6.57 ± 5.68		0.490	9.27 ± 5.55		6.31 ± 6.37		0.320
GC Cumulative Dose (g)	44.24 ± 52.26		15.65 ± 15.57		0.131	34.82 ± 42.84		16.77 ± 16.83		0.258
GC Intake per Day (mg)	30.62 ± 62.08		7.97 ± 9.51		0.284	20.44 ± 48.76		9.08 ± 10.32		0.501

Data are presented as mean ± standard deviation or as No. * *p*: *p*-value < 0.05. ^†^ Group 1: Glucocorticoid-induced cataract, Group 2: Age-related posterior subcapsular cataract, Group 3: Glucocorticoid-treated age-related cataract, Group 4: Normal control. Abbreviations: BCVA = best corrected visual acuity; GC = glucocorticoid; IPTW = inverse probability treatment weighting; logMAR = logarithm of the minimum angle of resolution; SE = spherical equivalent.

**Table 2:** miRNA expression in the study participants.

	ΔCt				*p*						
	Group ^†^ 1	Group 2	Group 3	Group 4	Among the Groups	Group 1 vs. 2	Group 1 vs. 3	Group 1 vs. 4	Group 2 vs. 3	Group 2 vs. 4	Group 3 vs. 4
let-7a-5p	−1.26 ± 4.12	3.02 ± 4.63	−1.68 ± 2.92	5.74 ± 1.88	0.000 *	0.094	0.826	0.000 *	0.064	0.262	0.000 *
let-7d-5p	−3.41 ± 6.25	−5.67 ± 6.07	−4.13 ± 6.65	−9.37 ± 1.90	0.013 *	0.519	0.828	0.015 *	0.696	0.243	0.096
let-7g-5p	−0.93 ± 1.41	−1.32 ± 1.48	−0.29 ± 1.40	−1.28 ± 1.21	0.363	0.635	0.383	0.574	0.262	0.959	0.161
miR-15a-5p	4.57 ± 6.32	0.86 ± 5.94	4.83 ± 6.07	−2.86 ± 0.82	0.002 *	0.297	0.935	0.005 *	0.296	0.227	0.018 *
miR-16-5p	2.40 ± 3.13	−0.46 ± 3.07	2.20 ± 2.69	−2.42 ± 1.86	0.000 *	0.119	0.898	0.001 *	0.152	0.165	0.001 *
miR-22-3p	1.53 ± 4.61	1.50 ± 5.55	3.38 ± 3.29	0.94 ± 0.65	0.638	0.992	0.393	0.699	0.500	0.834	0.113
miR-23a-3p	−2.52 ± 2.18	−2.03 ± 1.18	−2.01 ± 1.47	−1.47 ± 0.91	0.285	0.648	0.609	0.189	0.977	0.344	0.390
miR-23b-3p	−3.33 ± 2.22	−2.60 ± 0.24	−2.45 ± 1.91	−1.24 ± 0.78	0.045 *	0.329	0.421	0.017 *	0.850	0.000 *	0.172
miR-26a-5p	−1.62 ± 2.86	0.81 ± 2.54	−1.32 ± 1.91	4.58 ± 1.66	0.000 *	0.131	0.813	0.000 *	0.139	0.005 *	0.000 *
miR-34a-5p	5.13 ± 3.54	6.92 ± 5.13	3.85 ± 3.16	4.92 ± 1.52	0.791	0.453	0.468	0.872	0.248	0.439	0.386
miR-125a-5p	−1.96 ± 1.05	−2.02 ± 0.91	−1.46 ± 0.68	−0.26 ± 1.47	0.018 *	0.910	0.301	0.010 *	0.262	0.031 *	0.079
miR-125b-5p	−2.65 ± 1.13	−2.23 ± 1.44	−1.59 ± 1.28	−2.26 ± 0.72	0.534	0.554	0.100	0.383	0.450	0.963	0.211

Data are presented as mean ± standard deviation. * *p*: *p*-value < 0.05. ^†^ Group 1: Glucocorticoid-induced cataract, Group 2: Age-related posterior subcapsular cataract, Group 3: Glucocorticoid-treated age-related cataract, Group 4: Normal control weighting.

**Table 3:** Correlation between vital clinical characteristics and miRNA expression in glucocorticoid-induced cataracts.

	Age		GC Cumulative Dose		GC Intake per Day	
	r	*p*	r	*p*	r	*p*
let-7a-5p	−0.403	0.248	0.256	0.475	0.364	0.301
let-7d-5p	0.313	0.379	−0.216	0.549	−0.307	0.388
let-7g-5p	−0.240	0.505	0.122	0.738	0.388	0.268
miR-15a-5p	0.260	0.468	−0.194	0.592	−0.190	0.599
miR-16-5p	0.294	0.409	−0.228	0.526	0.035	0.925
miR-22-3p	−0.079	0.828	0.161	0.657	0.201	0.578
miR-23a-3p	−0.563	0.090	0.406	0.245	0.639	0.047 *
miR-23b-3p	−0.530	0.115	0.369	0.293	0.487	0.154
miR-26a-5p	−0.483	0.157	0.281	0.432	0.520	0.124
miR-34a-5p	0.081	0.824	−0.160	0.659	−0.084	0.817
miR-125a-5p	−0.569	0.086	0.283	0.428	0.688	0.028 *
miR-125b-5p	−0.287	0.422	0.003	0.994	0.544	0.104

* *p*: *p*-value < 0.05. Abbreviations: GC = glucocorticoid.

## Data Availability

The data presented in this study are available on request from the corresponding author. The data are not publicly available due to privacy restrictions.

## Abbreviations

The following abbreviations are used in this manuscript:

mRNA	microRNA
GIC	Glucocorticoid-induced cataracts
GC	Glucocorticoid
PSC	Posterior subcapsular cataract
ARC	Age-related cataract
qRT-PCR	quantitative real-time polymerase chain reaction
BCVA	Best-corrected visual acuity
LOCS	Lens Opacities Classification System
cDNA	complementary DNA
SD	Standard deviation
IPTW	Inverse probability of treatment weighting
ANOVA	One-way analysis of variance
logMAR	logarithm of the minimum angle of resolution
SE	Spherical equivalent
LEC	Lens epithelial cell
FGF	Fibroblast growth factors
TGF-β	Transforming growth factor-beta
